# Brain compensatory activation during Stroop task in patients with mild cognitive impairment: a functional near-infrared spectroscopy study

**DOI:** 10.3389/fnagi.2025.1470747

**Published:** 2025-02-07

**Authors:** Chenyu Fan, Hanfei Li, Ke Chen, Guohui Yang, Hongyu Xie, Haozheng Li, Yi Wu, Meng Li

**Affiliations:** ^1^Department of Rehabilitation Medicine, Huashan Hospital, Fudan University, Shanghai, China; ^2^State Key Laboratory of Transducer Technology, Shanghai Institute of Microsystem and Information Technology, Chinese Academy of Sciences, Shanghai, China; ^3^State Key Laboratory of Transducer Technology, University of Chinese Academy of Sciences, Beijing, China; ^4^INSIDE Institute for Biological and Artificial Intelligence, Shanghai, China

**Keywords:** mild cognitive impairment, fNIRS, executive function, Stroop task, cortical compensatory activation

## Abstract

**Purpose:**

This study investigated the disparities in brain activation patterns during the Stroop task among individuals with mild cognitive impairment (MCI) and those without any cognitive impairments (healthy controls, HCs) using functional near-infrared spectroscopy (fNIRS).

**Methods:**

We analyzed the cortical activation patterns of 73 patients with MCI and 63 HC individuals as they completed the Stroop task, employing fNIRS. The regions of interest (ROIs) included the dorsal prefrontal cortex (dPFC), ventrolateral prefrontal cortex (VLPFC), and parietal lobe (PL). The Stroop task is divided into early stage (0–15 s) and late stage (15–30 s). We also measured participants’ behavior during the Stroop task, analyzed variations in cortical activation intensity at different experiment stages, and performed correlation analysis between Montreal Cognitive Assessment (MoCA) scores, Stroop performance, and oxygenation levels.

**Results:**

Our analysis revealed that individuals with MCI and HC demonstrated elevated cortical activation in the dPFC, VLPFC, and PL areas while performing the Stroop task (*q* < 0.05, FDR-corrected). The MCI group displayed longer response latencies compared to the HC group while demonstrating comparable accuracy performance across both congruent and incongruent Stroop trials. The MCI group showed compensatory activation in the VLPFC, and PL regions compared to the HC group during the late stage of the Stroop task (*q* < 0.05, FDR-corrected). Correlational analysis revealed a negative association between MoCA scores and oxygenation levels in the dPFC, VLPFC, and PL regions during the late stage of the Stroop task (*p* < 0.05). However, no correlation was found with behavioral performance.

**Conclusion:**

Mild cognitive impairment patients demonstrated effective compensation for their cognitive impairments at a partial behavioral level by engaging compensatory activation in the prefrontal, and parietal regions while performing the Stroop task.

## Introduction

Mild cognitive impairment is identified by a decline in memory capabilities and other cognitive domains (Petersen et al., 1999), serving as an early sign of dementia (Scheltens et al., 2021). Patients with MCI experience notable deficits in executive functions (Corbo and Casagrande, 2022), impacting their ability to solve problems and control attention. As a result, they may encounter challenges when carrying out daily activities, making decisions, and adjusting to unfamiliar circumstances. This decline in cognitive function diminishes individuals’ quality of life and elevates the likelihood of advancing to Alzheimer’s disease or other forms of dementia (Kirova et al., 2015; Rattanavichit et al., 2022). Hence, it is crucial to identify executive function impairment in MCI patients early on for a more comprehensive comprehension and treatment of their condition.

The Stroop task is a valuable paradigm for investigating cognitive processing and executive functions by measuring the delay in reaction time between congruent and incongruent stimuli. Research using behavior and neuroimaging has shown that semantic interference results in inhibition during the Stroop task (Kirova et al., 2015; Rattanavichit et al., 2022; Scarpina and Tagini, 2017). The Stroop task aims to assess “inhibitory control (Stroop, 1935),” a facet of executive function (Miyake et al., 2000), which involves an individual’s capacity to handle cognitive interference and sustain attentional control. This task entails naming the color of words that may or may not semantically match, necessitating the use of executive control mechanisms to overcome automatic response inclinations and accomplish prioritized tasks (Miyake et al., 2000). Recent findings indicate that MCI is linked to a general deficit in inhibition (Rabi et al., 2020), the most prevalent and severe deficit among executive functions. As a result, employing the Stroop task with MCI patients holds promise for uncovering the underlying mechanisms of early neurodegenerative disease associated with cognitive decline (Kaufmann et al., 2008; Zurrón et al., 2018).

Contemporary studies suggest that the frontal-parietal network is considered essential for performing the Stroop task. The anterior cingulate cortex (ACC) is responsible for detecting conflicts in cognitive mechanisms related to the Stroop task, while prefrontal cortex is engaged in resolving these conflicts (Carter and van Veen, 2007). The oxygenation levels of ACC and prefrontal cortex reflect the degree of conflict detection and control (Era et al., 2021; Nee et al., 2007; Yin et al., 2021). PL activation may be associated with processing semantic conflicts and is thought to play a role in maintaining stimulus-response mappings relevant to the task (Bunge et al., 2002; Desmurget et al., 2018). Studies using functional magnetic resonance imaging (fMRI) have revealed that individuals with MCI exhibit notably elevated cortical activation in the dorsal anterior cingulate gyrus, bilateral middle and inferior frontal gyri, bilateral inferior parietal lobule, and bilateral insula during the Stroop task compared to HCs (Li et al., 2009). Moreover, researchers using fNIRS have indicated that both healthy controls and mild cognitive impairment patients engage the prefrontal cortex and parietal lobe when dealing with the intensity of a stimulus and interference while performing the Stroop task and MCI patients exhibit increased activation in central and thalamic regions compared to the HC group, potentially indicating a more demanding response selection process or inadequate inhibition control (Kaufmann et al., 2008). In previous research in the field of fNIRS, the prefrontal and parietal lobes were the focus areas of interest (Kaufmann et al., 2008). The findings of the present research are inconclusive, and there is still a need to explore the mechanism of blood oxygen activation in MCI patients during the performance of the Stroop task.

The brain activation patterns in individuals with MCI were significantly impacted by the neural compensation mechanism. The capacity for neural compensation is negatively correlated with the degree of degeneration in neural systems (Price and Friston, 2002). There is growing evidence indicating that individuals with MCI may require the involvement of extra brain regions to perform tasks, which is seen as a form of compensatory mechanism (Kaufmann et al., 2008; Li et al., 2009; Pinti et al., 2020). Neural compensation refers to the compensatory activation related to aging and neural activity, especially in the prefrontal cortex. Previous studies have observed compensatory activation in both hemispheres of the prefrontal cortex in elder adults when engaging in challenging task paradigms (Cappell, 2010; Orth et al., 2024; Shatz, 2024). In cognitive aging theory, the concept of scaffolding refers to the compensatory activation in the prefrontal and parietal lobes of aging individuals, particularly highlighting frontal recruitment. This mechanism effectively mitigates the effects of brain degeneration on cognitive functioning (Reuter-Lorenz and Park, 2014). Cognitive reserve could stem from the more efficient utilization of brain networks or the enhanced capacity to engage alternative brain networks as required (Stern, 2002). The most appropriate cognitive aging theory for patients with MCI remains a topic of debate and further research evidence is required to support detailed studies in this area.

Functional near-infrared spectroscopy is a non-invasive method of optical imaging that can measure the levels of oxygenated hemoglobin associated with brain activity (Pinti et al., 2020). The timing and pattern of hemodynamic response recorded by fNIRS are comparable to those observed in the blood oxygenation-dependent (BOLD) signal obtained from fMRI (Grässler et al., 2021; Yang et al., 2019; Yoo et al., 2020), making it a promising tool for studying brain function. Due to its portability, relatively low cost, and ability to accommodate minor movements, fNIRS is especially well-suited for clinical environments. Its research outcomes could be utilized for cognitive assessments in older adults and individuals with cognitive impairments (Butters et al., 2023). Previous fNIRS research has primarily concentrated on the prefrontal cortex due to limitations in signal collection caused by hair coverings (Grässler et al., 2021; Yang et al., 2019; Yang et al., 2020; Yoo et al., 2020; Yoon et al., 2019). A prior study broadened the uses of fNIRS by pinpointing the frontal and parietal lobes as ROIs and noting a decrease in connectivity between the frontal and frontoparietal areas in individuals with Parkinson’s-related MCI (Shu et al., 2024). Consequently, broadening the application of fNIRS to encompass additional brain regions can facilitate the exploration of pathological mechanisms underlying abnormal brain activation in MCI patients, specifically the compensatory mechanism, thereby increasing its diagnostic significance for clinical cognitive screening purposes.

In this study, we utilized behavioral performance, as well as cognitive impairment. Consistent with the theory of neural compensation, our analysis indicated evidence of neural compensation in MCI patients for maintaining task performance. This study is believed to be one of the first multi-regional fNIRS studies evaluating brain activation patterns in both MCI patients and HC while performing the Stroop task.

## Materials and methods

### Participants

A total of 73 MCI patients were selected from the Xujiahui Community Health Service Center, and 63 gender- and age-matched HC individuals were recruited from the local community for this research. The criteria for MCI, as defined by Petersen et al. (1999), include the following: (1) self-reported or informant-noted cognitive decline; (2) objective evidence of memory or other cognitive impairments 1–2 standard deviations below the norm for age and education; (3) unaffected daily living abilities; (4) exclusion of a dementia diagnosis. All participants and the control group ranged in age from 60 to 85 years, had over 6 years of education, and did not exhibit any notable visual or hearing deficiencies. There were no indications of major psychiatric disorders, other significant neurological conditions, or cerebrovascular diseases among the participants. Demographic information for all subjects, including age, gender, height, weight, education level and MoCA scores, was provided in Table 1. The study protocol was approved by the Ethics Committee of Huashan Hospital, Fudan University. Prior to the experiment, all participants were thoroughly informed about the experimental procedures and provided their written informed consent. Additionally, this trial received approval from the Clinical Research Information Service (CRIS) of China, a publicly accessible primary register that is part of the WHO International Clinical Trial Registry Platform. The trial was registered on 06 March 2022, with registration number ChiCTR2200057281.

**TABLE 1:** Participants’ demographic information.

Characteristic	MCI (*n* = 73)	HC (*n* = 63)	*P*-value
Age (years)	74.51 ± 6.82	74.18 ± 6.02	0.77
Gender (male/female)	27/44	28/35	0.24
Height (cm)	162.31 ± 7.96	162.93 ± 7.05	0.64
Weight (kg)	63.02 ± 9.03	63.27 ± 9.26	0.88
Education level (years)	11.23 ± 3.21	11.73 ± 2.92	0.33
MoCA	20.73 ± 3.55	26.28 ± 1.90	**0.00**
HLVT-R-learning	13.49 ± 4.21	17.62 ± 5.42	**0.00**
HLVT-R-memory	2.72 ± 2.45	5.63 ± 2.64	**0.00**
VDT-functional association	3.39 ± 0.76	3.67 ± 0.63	**0.00**
VDT-semantic association	2.81 ± 0.95	3.27 ± 0.68	0.21
VDT-visual recognition	5.52 ± 1.43	5.73 ± 1.54	0.49
VDT-visual reasoning	4.47 ± 2.21	6.51 ± 2.13	**0.00**
DST-forward	6.32 ± 3.94	7.43 ± 4.44	0.45
DST-backward	3.63 ± 2.65	4.95 ± 3.22	**0.00**
VFT-vegetable	13.55 ± 3.37	14.59 ± 3.72	0.53
VFT-fruit	9.27 ± 2.64	10.67 ± 3.54	**0.00**
VFT-idiom	3.08 ± 2.76	5.67 ± 3.23	**0.00**

The HVLT-R stands for the Hopkins Verbal Learning Test-Revised. The abbreviation for the Visual Discrimination Test is VDT, the Digital Span Test is DST, and the Verbal Fluency Test is VFT. The bold value of *p* is less than 0.05.

### Stroop task

**FIGURE 1:**
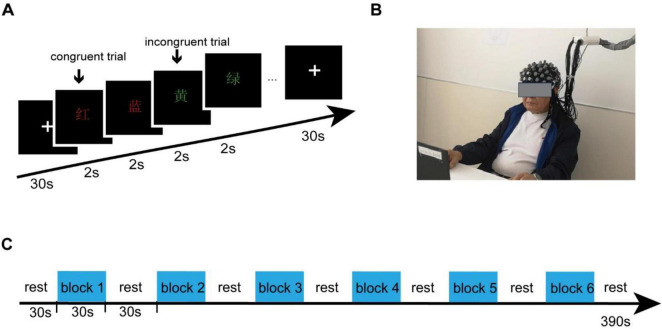
Experiment procedures. **(A)** The Stroop task consisted of four Chinese characters - “RED,” “YELLOW,” “GREEN,” and “BLUE” - presented in different colors at the center of the screen. **(B)** Participants were equipped with fNIRS sensors and positioned in front of a monitor as part of the experimental setup. **(C)** The task continued for a total of 390 s and was split into six segments, each consisting of a 30 s task followed by a 30 s break. Participants were instructed to press the red button with their right hand for a congruent stimulus, and the yellow button with their left hand for an incongruent stimulus.

This research utilized the Stroop task with four Chinese characters: “RED,” “YELLOW,” “GREEN,” and “BLUE.” These characters were presented in various colors at the center of the screen. Participants were required to promptly and precisely determine if the color matched the meaning of the Chinese character. The task lasted for a total of 390 s and was structured into six segments. There is a 30 s break at the beginning of the task, each segment consists of a 30 s task followed by a 30 s break period. Throughout each task, individuals were exposed to 15 stimuli in each segment - comprising five congruent stimuli and 10 incongruent ones, with each stimulus being displayed for a duration of 2 s as illustrated in Figure 1. Upon arriving at the research laboratory, participants receive instructions on the operational procedures of fNIRS and the Stroop paradigm. To guarantee high-quality data collection from the fNIRS equipment and help participants adapt to the fNIRS headcap, we offer them the chance to practice the Stroop paradigm using the e-prime software while wearing the fNIRS headcap. Participants were instructed to press the red button with their right hand for a congruent stimulus, and the yellow button with their left hand for an incongruent stimulus. E-Prime 3.0 software was used to record accuracy rates and reaction times.

### fNIRS recording

**FIGURE 2:**
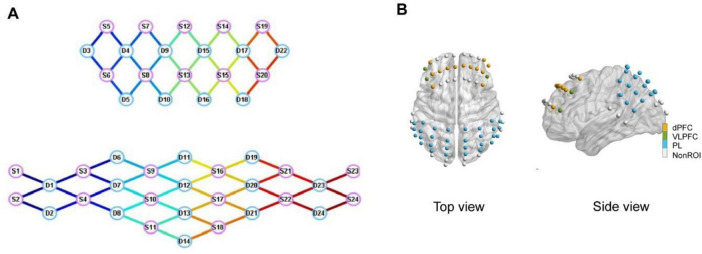
**(A)** Topology of functional near-infrared spectroscopy (fNIRS) sensors. S1–S24 denote the sources and D1–D24 denote the detectors. **(B)** Spatial distribution of a total of 70 fNIRS sensors on the cerebral cortex.

A multi-channel fNIRS imaging device (NirScan-8000, DanYang HuiChuang Medical Equipment Co., Ltd., China) was employed to collect data from participants while they performed the Stroop task, detecting alterations in HbO signals. The sampling rate for all channels was configured at 11 Hz, utilizing wavelengths of 730 and 850 nm. A special fNIRS headcap was created to fulfill the requirements of the experiment, tailored according to the participants’ characteristics and adhering to the widely recognized 10/20 electrode placement system. In total, 48 probes (24 sources and 24 detectors) were positioned throughout the entire cortex with a spacing of 30 mm between each probe, forming a network of 70 channels as shown in Figure 2. The channel positions were established using the Patriot digitizer (Polhemus, United States) on a standard head model and then standardized to the Montreal Neurological Institute (MNI) space. The MNI coordinates were visualized using the BrainNet Viewer toolbox within MATLAB (2022B), utilizing the BrainMesh_ICBM 152 template. Subsequently, the MNI coordinate of each channel was assigned to specific brain regions based on the Broadmann Talairach template.

Previous research led us to categorize the prefrontal cortex into specific areas of interest, including the dPFC and VLPFC (Yoo et al., 2020). Additionally, the fNIRS headcap also encompassed the PL regions. The comprehensive breakdown of ROIs is provided in Table 2.

**TABLE 2:** Regions of interest (ROIs) segmentation.

ROIs	Channels
dPFC	12, 14, 15, 18, 20, 35, 37, 41, 43,56, 57, 58, 59
VLPFC	11, 13, 60
PL	4, 5, 6, 7, 8, 9, 10, 22, 23, 24, 25, 26, 27, 28, 29, 45, 46, 47, 48, 49, 50, 51, 52, 61, 62, 63, 64, 65, 66, 67

### Data preprocessing

The data preprocessing was carried out in MATLAB using a pipeline based on the HOMER2 toolbox (Huppert et al., 2009). First, the original light intensity signal underwent a conversion to an optical density (OD) signal. Next, a motion correction algorithm was implemented utilizing a sliding window approach for each channel independently. If within each 1 s window, the portion of the OD signal showed deviation from the mean by more than five times the standard deviation, it was identified as containing motion artifacts, and the corresponding OD signal was discarded and reconstructed using the spline interpolation method. Following this step, the OD signal was converted into concentrations of HbO via the application of the modified Beer-Lambert law (Scholkmann et al., 2014). Finally, physiological noises including respiration, cardiac activity and low-frequency drift signals were eliminated through digital bandpass filtering employing a Butterworth filter in frequencies ranging between 0.01 and 0.1 Hz.

### Data analysis

The preprocessed data involved 136 participants, with 70 channels for each participant, and every channel consisting of six blocks. The signal-to-noise ratio (SNR) of each channel was assessed by calculating the coefficient of variation (CV) at the block level for each signal channel as follows:


$$CV_{b\,l\,o\,c\,k}\left(\%\right)=\frac{\sigma_{b\,l\,o\,c\,k}}{\mu_{b\,l\,o\,c\,k}}\times 100\%$$

Where σ_*block*_ and μ_*block*_ denoted the standard deviation and the mean of a 30 s block of the Stroop task. If a block’s CV surpassed 0.1 (Lu et al., 2015), the signal from that block was considered unreliable and eliminated. Additionally, if over half of the blocks for a specific channel were excluded, then the signal for that channel was also dismissed.

The channel-level CV was calculated as follows:


$$CV_{c\,h\,a\,n\,n\,e\,l}\left(\%\right)=\frac{\sigma_{c\,h\,a\,n\,n\,e\,l}}{\mu_{c\,h\,a\,n\,n\,e\,l}}\times 100\%$$

Where σ_*channel*_ and μ_*channel*_ were the standard deviation and the mean of a 390 s Stroop’s channel. If a channel’s CV surpassed 0.15 (Piper et al., 2014), that channel’s signal was eliminated. Additionally, if over 10% of the channels within a subject’s dataset were removed, the entire data for that subject was rejected.


$$M\,e\,a\,n_{c\,h\,a\,n\,n\,e\,l}=\frac{M\,e\,a\,n_{s\,i\,g\,n\,a\,l}-M\,e\,a\,n_{b\,a\,s\,e}}{M\,e\,a\,n_{b\,a\,s\,e}}$$

In this calculation, *Mean*_*signal*_ represented the mean signal value of the HBO during the time range from 0 to 60 s. *Mean*_*base*_ denoted the baseline mean value measured from -2 to 0 s prior to the onset of the task or stimulus. This computation effectively quantified the relative change in HbO concentration from the baseline to the active state, normalized to the baseline level.

Four brain regions, including the dPFC, VLPFC, and PL, were selected as ROIs for this study. The mean value of ROI was calculated as follows:


$$M\,e\,a\,n_{R\,O\,I}=\frac{\Sigma \,M\,e\,a\,n_{c\,h\,a\,n\,n\,e\,l}}{N}$$

Where Σ*Mean*_*channel*_ denoted the sum of *Mean*_*signal*_ of the selected channels, *N* was the number of channels of the ROIs, as indicated in Table 2. The *Mean*_*ROI*_ represented the level of brain activation intensity.

The pre-processed time-series data of HbO for each participant’s ROIs underwent analysis using the generalized linear model (GLM). The GLM generated an optimal hemodynamic response function (HRF) for each experimental condition and participant and then evaluated the consistency between the observed experimental HRF values and the ideal ones. By fitting experiment data to specific setups, the GLM calculated an activation coefficient beta value. This beta value, which represents the peak of the HRF function, indicates the level of cortical activation in response to the experiment within a brain region. Typically, beta values that reflect peak HRF functions were used in predicting estimates for HbO signals to demonstrate levels of cortical activation. Sets of beta values from each brain region signified cortical activation levels.

### Lateralization of brain function

The calculation of Lateralization involved the use of an eigenvalue index based on the average blood oxygen levels, represented by the Lateralization index (LI) (Vernooij et al., 2007). Prior research showed that LI ≥ 0.1 indicated left Lateralization, while LI ≤ −0.1 indicated right Lateralization.


L⁢I=M⁢e⁢a⁢nR⁢O⁢Il⁢e⁢f⁢t-M⁢e⁢a⁢nR⁢O⁢Ir⁢i⁢g⁢h⁢tM⁢e⁢a⁢nR⁢O⁢Il⁢e⁢f⁢t+M⁢e⁢a⁢nR⁢O⁢Ir⁢i⁢g⁢h⁢t

where M⁢e⁢a⁢nR⁢O⁢Il⁢e⁢f⁢t denoted the average signal mean of the HbO within the left ROIs. M⁢e⁢a⁢nR⁢O⁢Ir⁢i⁢g⁢h⁢t represented the average signal mean of the HbO within the right ROIs.

### Statistical analysis

Basic demographic information such as age, height, weight, the MoCA score, and education level, as well as participants’ behavioral performance in terms of reaction times and accuracy in congruent and incongruent conditions, were evaluated using independent sample *t*-tests. Gender differences were examined using chi-square tests.

The Shapiro-Wilk test was used to evaluate the normality of average HbO concentration within each ROI, confirming its adherence to a normal distribution. Independent sample *t*-tests were conducted to evaluate differences in mean HbO levels within ROIs and LI of the MCI and HC groups. Furthermore, we computed the effect size for the independent-sample *t*-test. To mitigate false positives, a significance threshold of *q* ≤ 0.05 was established and False Discovery Rate (FDR) correction was implemented for the statistical results regarding average HbO concentration within ROIs.

A Pearson correlation analysis was conducted with the dual aims of investigating the interplay between MoCA scores and the average concentration of HbO within specified ROIs, as well as scrutinizing the correlational patterns that exist between behavioral performance indicators and blood oxygen levels.

## Results

### Behavioral analysis

We first examined whether there were behavioral differences between the MCI and HC groups when performing the Stroop task. The response times and accuracy were measured for each trial during the experiment. The results for both the MCI and HC groups are presented in Table 3. According to researcher feedback, all participants became familiar with the paradigm content after a single practice session and did not request additional practice. Furthermore, we examined the distribution of accuracy and found that the accuracy rates of both groups were above 60%, with no extreme values observed. This indicates that the participants accurately understood the content of the Stroop task and made appropriate choices within their abilities. Our analysis revealed no statistically significant variances in total reaction time and accuracy between the two groups. We further investigated the distribution of reaction time and accuracy in the MCI group and HC group, categorized into congruent and incongruent Stroop trials. The findings demonstrated no substantial difference in accuracy between the two subject groups in the Stroop task, but a discrepancy in the distribution of reaction time. Notably, the response time of the MCI group was significantly prolonged compared to the HC group in both congruent and incongruent Stroop trials (*p* < 0.05).

**TABLE 3:** Analysis of behavioral performance.

	MCI (*n* = 73)	HC (*n* = 63)	*P*-value
Reaction time (ms)	985.51 ± 99.81	947.64 ± 139.43	0.06
Congruent	976.43 ± 101.32	910.32 ± 1.34	**0.03**
Incongruent	1099.32 ± 100.32	976.51 ± 110.03	**0.04**
Accuracy (%)	90.21 ± 13.76	87.95 ± 11.02	0.30
Congruent	91.22 ± 15.66	88.95 ± 13.05	0.34
Incongruent	89.43 ± 14.87	89.45 ± 15.32	0.45

The bold value of *p* is less than 0.05.

### Brain activation

**FIGURE 3:**
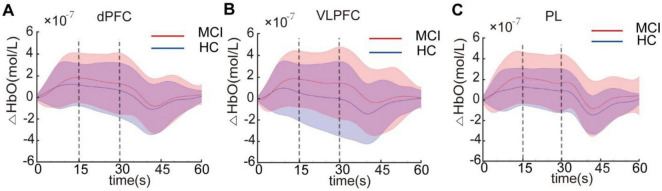
The average HbO concentration in the dorsal prefrontal cortex (dPFC) **(A)**, ventrolateral prefrontal cortex (VLPFC) **(B)**, and parietal lobe (PL) **(C)** for the mild cognitive impairme (MCI) and healthy controls (HC) groups. Each Stroop task was divided into the early stage (0–15 s) and late stage (15–30 s).

We then investigated potential disparities in brain activity between the MCI and HC groups during the administration of the Stroop task. The brain’s activation state was determined by examining the average HbO concentration during the Stroop task in both the MCI and HC groups. Previous research on hemodynamic activation in young adults revealed a peak response approximately 5 s after stimulus onset (Pinti et al., 2020). This represents a typical blocked design employed in near-infrared spectroscopy tasks, where participants undergo cognitive activities for 30 s, triggering the activation of corresponding brain areas, after which they have a 30 s rest interval to allow their brains to revert to a resting condition. This is a classic block design for tasks conducted under near-infrared spectroscopy, where participants engage in cognitive responses during the 30 s task period, activating corresponding brain regions, followed by a 30 s rest period allowing the brain to return to a resting state. However, as shown in Figure 3, our study found a slower hemodynamic response in HCs than in the MCI group. During the early stage of the Stroop task (0–15 s), the hemodynamic response continued to increase, while in the later phase (15–30 s), it stabilized at a relatively constant level for both groups. This may reflect the distinct hemodynamic characteristics of MCI patients and HCs at different stages of the Stroop task (Yang et al., 2020). Figure 3 displayed the average HbO curves for ROIs, including the dPFC, VLPFC, and PL, during the early and late stages of the Stroop task. Generalized linear model analysis indicated that the dPFC, VLPFC, and PL were all significantly activated (*q* < 0.05) in both the early and late stages, suggesting these brain regions were involved in the cognitive processes of the Stroop task.

**FIGURE 4:**
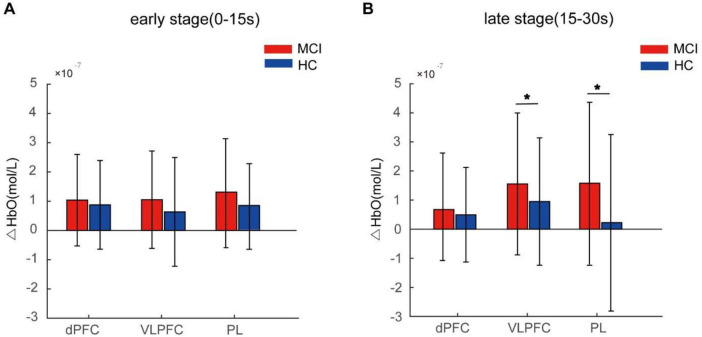
Average HbO concentration of the mild cognitive impairme (MCI) and healthy controls (HC) groups during the early stage (0–15 s) (**A**) and the late stage (15–30 s) (**B**). Error bars represent standard error. The asterisk denotes *q* < 0.05.

Intra-group statistical analysis showed activation in the dPFC, VLPFC, and PL areas during the Stroop task for both participant groups after FDR correction (*q* < 0.05). Inter-group statistical analysis revealed no significant difference in activity levels among these brain regions at the early stage between the two participant groups. However, during the late stage, MCI patients exhibited significantly increased activation in the VLPFC, and PL brain regions compared to HC (*q* < 0.05). There was no significant difference observed in the dPFC region as shown in Figure 4.

We calculated Cohen’s d to assess the effect sizes of brain activation between MCI and HC groups (Westfall et al., 2014; Reddy et al., 2021). Cohen’s d values of 0.2, 0.5 and > 0.8 denote small, medium and large effects, respectively (Sawilowsky, 2009). Lower effect sizes were observed in dPFC, VLPFC, and PL during the early stage compared to the late stage in Table 4, indicating more pronounced disparities between MCI and HC groups in the late stage, which aligns with the findings presented in Figure 4.

**TABLE 4:** Effect size of the brain activation of the mild cognitive impairment (MCI) and healthy controls (HC) groups.

	Early stage	Late stage
dPFC	0.10	0.26
VLPFC	0.24	0.47
PL	0.27	0.36

**FIGURE 5:**
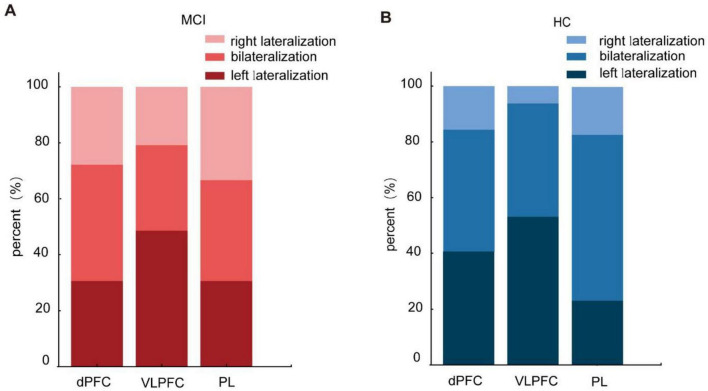
Lateralization of activated brain regions in the Stroop task of the mild cognitive impairme (MCI) (**A**) and healthy controls (HC) (**B**).

Lateralization analysis was carried out on the dPFC, VLPFC, and PL brain regions of two groups to examine the laterality of brain activation patterns during the Stroop task. The statistical analyses revealed no significant differences in brain lateralization patterns between the MCI and HC groups during the Stroop task. Previous research indicated that the LI for left lateralization was ≥ 0.1, while the LI for right lateralization was ≤ -0.1 (Vernooij et al., 2007). In this study, we examined the LI at the individual level, and the results are presented in Figure 5. Our individual-level analysis revealed substantial variability in lateralization within both the MCI and HC groups. Consequently, caution is warranted when drawing conclusions about cerebral lateralization based on individual-level findings.

### Correlation analysis between brain activation and MoCA scores

Next, to determine whether the average values of the ROIs could distinguish between the MCI and HC groups as assessed by MoCA scores, a correlation study was carried out. Metric values and MoCA scores from both groups were combined into a single dataset. A Pearson correlation analysis was conducted to examine the relationship between these parameters, where the metric values represent the mean HbO concentration. This analysis yielded the Pearson product-moment correlation coefficient. The results showed that in the early stage, there was no significant correlation between mean HbO concentration and the MoCA scores across both participant groups.

**FIGURE 6:**
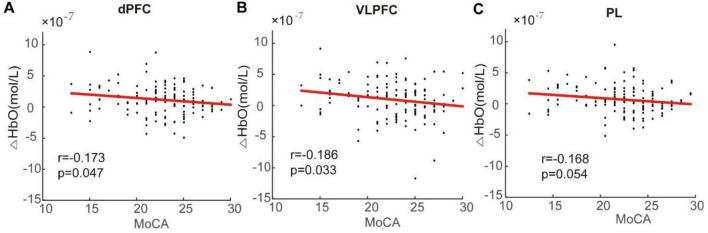
Correlation analysis between change in oxygenation level and Montreal Cognitive Assessment (MoCA) score in the dorsal prefrontal cortex (dPFC) **(A)**, ventrolateral prefrontal cortex (VLPFC) **(B)**, and parietal lobe (PL) **(C)**. Negative correlations were observed between the mean HbO concentration and MoCA scores. Specifically, decreased activation levels in the dPFC, VLPFC, and PL were associated with lower MoCA scores.

During the late stage, however, a significant negative correlation was identified between average HbO concentration and MoCA scores. Specifically, there were negative correlations observed in the activation intensity of dPFC (*r* = -0.173, *p* = 0.047) and VLPFC (*r* = -0.186, *p* = 0.033) regions concerning MoCA scores. Furthermore, there was an almost significant correlation coefficient for the average HbO concentration in the PL region (*r* = -0.168) at a level approaching significance (*p* = 0.054), as shown in Figure 6. There was no substantial relationship observed between the average HbO concentration in the late stage and behavioral parameters in either group of participants.

## Discussion

In the present study, we examined the cortical activation patterns in MCI patients and HC individuals while performing the Stroop task. The study revealed that MCI patients demonstrated effective compensation through increased activation in the prefrontal cortex and parietal lobe to achieve comparable behavioral performance. These findings offer support for the relevance of neural compensation theory in understanding cognitive impairments (Price and Friston, 2002).

Previous research has indicated that the MCI group displayed difficulties with inhibitory control, leading to extended reaction times and decreased accuracy rates in comparison to the HC group (Cappell, 2010). However, a separate study discovered that individuals with MCI still retained some capacity for goal maintenance, enabling them to sustain comparable error rates by slowing down their response speeds (Bélanger et al., 2010). Our results from the neuropsychological scale showed that individuals with MCI achieved significantly lower scores on scales related to executive function in comparison to the HC group. This outcome might be affected by the recruitment procedure. Our study recruited residents from the community for evaluations using neuropsychological scales and then chose participants based on these assessment results. Consequently, prior to undertaking the Stroop task, participants were already informed about their cognitive assessment results and clinical diagnosis. Notably, while MCI patients and HCs exhibited comparable accuracy on the Stroop task, the MCI patients displayed longer response times for both congruent and incongruent Stroop trials. This suggests that compared to HC individuals, those with MCI may demonstrate a heightened focus on their performance during the Stroop task and may prioritize accuracy over speed, achieving effective compensation at a behavioral level.

Our specially designed headcap offers us an opportunity to examine cortical activity in different areas of the brain while conducting the Stroop test. As shown in Figure 3, both MCI patients and HC exhibited significant cortical activation in the prefrontal cortex and parietal lobe while performing the Stroop task. Previous studies have indicated that the Stroop task primarily elicited activation in the frontal and parietal regions of the brain (Stojan et al., 2023). The prefrontal cortex is believed to be associated with executive functions, which oversee cognitive and behavioral processes (Zainal and Newman, 2018). In older adults, the prefrontal lobe is considered a critical area that offers compensatory resources to handle growing task demands and offset capacity compensation to other parts of the brain (Lamichhane et al., 2020). Previous research has indicated that the prefrontal cortex is associated with conflict processing (van Veen and Carter, 2005), while the activations in VLPFC may play a significant role in differentiating MCI patients from HC (Yoo et al., 2020). Yang et al. (2019) employed machine learning techniques and concluded that classifying the prefrontal cortex into sub-regions yielded better results than treating it as a singular unit. As such, our study subdivided the prefrontal cortex into dPFC and VLPFC for analysis. The parietal lobe covers areas 39 and 40 of Brodmann’s division, also known as Wernicke’s area, crucial for information integration and processing. Previous studies have shown that the compensatory increases in cortical activation tend to extend to the parietal region due to connectivity between prefrontal and parietal areas when HCs perform challenging tasks (McDonough et al., 2022). Future research should involve the careful selection of relevant brain areas based on the age and characteristics of the subjects. It is important to focus on compensatory activation in brain regions beyond the prefrontal cortex when elderly adults and patients with MCI perform cognitive tasks. Therefore, this study emphasized multi-brain region research using fNIRS, which represents a crucial research direction within this field.

Our results showed that there was no difference in the level of cortical activation between patients with MCI and HCs during the early stage of the Stroop task. In the late stage, however, patients with MCI demonstrated compensatory activation in brain regions such as VLPFC, and parietal lobe compared to the HC group. And we found the effect sizes were larger in the late stage. Despite no notable divergence in behavioral performances between both groups, our findings indicated that MCI patients showed effective compensation through additional activation in these brain areas. However, the findings of similar studies in the literature show some inconsistency. Li et al. (2009) showed that MCI patients exhibited significantly increased activation in several brain regions including the dorsal anterior cingulate gyrus, bilateral middle and inferior frontal gyri, bilateral inferior parietal lobules, and bilateral insula during the Stroop task compared to HCs. Another study discovered that both MCI patients and HC exhibited substantial activation in the intra-parietal and prefrontal regions, indicating that both groups utilize similar brain areas when managing the intensity of processing and interference (Kaufmann et al., 2008). The research conducted by Yoo et al. (2020) revealed that the VLPFC region was activated in healthy older individuals while performing the Stroop task, but this activation was not observed in MCI patients. Some studies found no differences in brain pattern activation during the Stroop task between MCI and HC (Puente et al., 2014; Yoo et al., 2020). The inconsistency in the prior study findings could be linked to the level of cognitive decline in MCI, sample sizes, and the specific cortical areas under examination. As a result, future explorations may involve standardizing the severity of MCI among participants, increasing enrollment numbers, and expanding research into wider brain regions for detection.

Moreover, lateralization of the left and right hemispheres was also a frequent concern in the Stroop task. In this study, activation patterns across individual brain regions were calculated at the group and individual levels. The results found that there were no notable variations in brain lateralization patterns between the MCI and HC groups while performing the Stroop task, but a high variability of LI was found at the individual level. The study found that HC and non-amnestic MCI patients were overactive in the right prefrontal area with predominant lateralization during the Stroop task in the forgotten MCI group (Yoon et al., 2019). Subgroup analysis of MCI was not performed in this study, and exploring the brain lateralization patterns of different types of MCI was well worth exploring in the future.

Our analysis noted that during the early stage of the Stroop task, there was no correlation between brain activity and the MoCA score. However, in the late stage, a negative correlation was observed between the intensity of activation in dPFC and VLPFC regions with the MoCA score. Also, the correlation coefficient for activation intensity in the parietal lobe and MoCA score almost reached statistical significance. In addition, our results indicated that there was no relationship between the level of brain region activation and task performance during the Stroop task for both MCI and HC groups. All aforementioned results indicate that individuals with lower cognitive levels exhibit higher compensatory activation in the prefrontal cortex and parietal lobe, aligning with inter-group comparison results.

Mild cognitive impairmeni is frequently observed among older adults, and neuroimaging studies have linked changes in cortical activation patterns to age-related alterations in neural activity. Early theories of neurocognitive aging based on structural MRI emphasize the frontal lobe hypothesis, which suggests that atrophy begins in the prefrontal cortex as early as middle adulthood. This cerebral degeneration can subsequently contribute to diverse cognitive impairments (West, 1996). Individuals experiencing aging and cognitive decline often display anatomical alterations, such as cortical atrophy. However, these individuals also exhibit enhanced neural activation in the corresponding brain areas, particularly the prefrontal cortex, during the successful execution of cognitive tasks. This heightened neural activity is interpreted as a mechanism of neural compensation (Cabeza, 2002; Reuter-Lorenz and Cappell, 2008). Previous research has shown that the connectivity between the PFC and lateral parietal cortex may lead to a compensatory increase that extends to the medial temporal lobe and parietal lobe (Dosenbach et al., 2008; Huang et al., 2012; Nagel et al., 2009; Vincent et al., 2008). The scaffolding theory of aging and cognition (Reuter-Lorenz and Park, 2014) proposes that connections between the frontal and parietal lobes can mitigate or offset the adverse effects of cognitive decline and neural impairment. Researchers have proposed that the observed changes in brain activation patterns among MCI patients completing cognitive tasks may be explained by the concept of neural compensation, this theory suggests that the brain adaptively reorganizes its neural networks to compensate for the functional changes associated with MCI (Ung et al., 2020). However, the specific patterns of neural compensation in MCI patients during Stroop tasks have not been extensively investigated and require further research to confirm. Previous research using fMRI has shown that during Stroop tasks, individuals with MCI exhibited compensatory activation in the prefrontal cortex and parietal cortex compared to healthy older adults (Li et al., 2009). Additionally, increased activation was observed in deep brain regions in the MCI patients. Furthermore, Kaufmann et al. (2008), also utilizing fMRI, found that MCI patients demonstrated higher activation intensities in the anterior and posterior cingulate cortices as well as the thalamus compared to neurologically normal older adults, suggesting the presence of neural compensation mechanisms in MCI. Prior research using fNIRS has not identified statistically significant differences in brain activation patterns between MCI patients and cognitively healthy older adults during the performance of Stroop tasks (Yoo et al., 2020; Yoon et al., 2019). Through intragroup analysis, studies have shown that cognitively healthy older adults activated the ventrolateral prefrontal cortex region during Stroop tasks, while MCI patients did not exhibit this activation (Yoo et al., 2020). Additionally, both MCI patients and cognitively healthy older adults demonstrated increased activation in the right prefrontal cortex during Stroop tasks (Yoon et al., 2019). Researchers have employed machine learning techniques to analyze brain activity patterns during Stroop task performance, identifying relevant biomarkers that may guide future research in this domain (Grässler et al., 2021; Shu et al., 2024; Yang et al., 2019; Yang et al., 2020). However, there remains limited empirical evidence on the hemodynamic activation mechanisms underlying the Stroop task responses of MCI patients assessed using fNIRS. Our study independently examined the brain activation patterns before and after completing the Stroop task, identifying statistically significant differences that validate the neural compensation theory. Moreover, the correlation analysis revealed an inverse relationship between MoCA scores and activation in the frontal, and parietal regions across both groups. This finding suggests that in MCI patients, compensatory brain activation is inversely proportional to the degree of cognitive deficits. However, the extent to which this phenomenon can be generalized to a broader population with varying levels of cognitive impairment requires further validation through studies with larger sample sizes.

Despite uncovering several original discoveries in our research, there remain several significant potential constraints. First, the status of MCI could produce varying outcomes. MCI represents an ongoing condition of cognitive dysfunction, with some participants in this research from community settings displaying mild impairment, while others recruited by different researchers from hospitals exhibited more severe impairment. The degree of cognitive dysfunction observed in cases of MCI is strongly related to the brain’s capacity for compensatory activation. Nevertheless, the specific threshold at which compensatory activation becomes critical for cognitive impairment and task difficulty had not been established and necessitated further investigation. Secondly, MCI can be categorized into amnestic and non-amnestic subtypes based on the specific cognitive functions affected. The amnestic subtype involves impairment in memory, while the non-amnestic subtype involves deficits in other cognitive domains with relatively preserved memory. Our investigation did not distinguish between these MCI subtypes, aiming to elucidate the brain activation characteristics of MCI patients in a community setting. However, the varying cognitive impairments in different MCI subtypes result in disparities in behavioral performance on the Stroop task, which represents a limitation of our study. Future research endeavors should focus on cognitive assessments using fNIRS tailored to different MCI subtypes, as well as analyzing the correlation between the impaired cognitive domains, behavioral performance, and brain activation patterns across various MCI subtypes. Thirdly, the selection of ROIs and the cortical area covered by the fNIRS headcap could influence the outcomes. Part of the reason why no significant activation difference was detected in dPFC, might be that far more sensors were included, and averaging across so many different sensors may be obscuring an effect that’s more spatially selective. Fourthly, according to the channel arrangement of the fNIRS headcap, the sensor coverage of the VLPFC was quite limited. In future research, more probes or headcap combining long and short source-detector separation can be used to explore specific brain regions. Finally, before implementing the research findings in clinical settings, it may be essential to carry out several fNIRS-based studies on the same subject to assess reliability and validity.

## Conclusion

In summary, MCI patients exhibited partial behavioral compensation for their cognitive impairments by activating compensatory mechanisms in the prefrontal, and parietal regions during the Stroop task. Our results provide further support for the neural compensation theory, indicating that MCI patients may compensate for cognitive impairments by additionally activating brain regions such as the prefrontal cortex and PL during the Stroop task to achieve behavioral compensation.

## Data Availability

The raw data supporting the conclusions of this article will be made available by the authors, without undue reservation.
